# Case Report: Exceptional Response to Nivolumab Plus Ipilimumab in a Young Woman With TFE3-SFPQ Fusion Translocation-Associated Renal Cell Carcinoma

**DOI:** 10.3389/fonc.2021.793808

**Published:** 2021-12-16

**Authors:** Dylan J. Martini, Caroline S. Jansen, Lara R. Harik, Sean T. Evans, T. Anders Olsen, Viraj A. Master, Haydn T. Kissick, Mehmet Asim Bilen

**Affiliations:** ^1^ Department of Medicine, Massachusetts General Hospital, Boston, MA, United States; ^2^ Department of Hematology and Medical Oncology, Winship Cancer Institute of Emory University, Atlanta, GA, United States; ^3^ Department of Urology, Emory University School of Medicine, Atlanta, GA, United States; ^4^ Department of Pathology, Emory University School of Medicine, Atlanta, GA, United States; ^5^ Department of Hematology and Medical Oncology, Emory University School of Medicine, Atlanta, GA, United States

**Keywords:** translocation-associated RCC, combination immune checkpoint therapy, exceptional responder, intratumoral immune niche, TCF1+ CD8+ T cells, rare malignancy

## Abstract

Translocation-associated renal cell carcinoma (tRCC) is a rare, aggressive malignancy that primarily affects children and young adults. There is no clear consensus on the most effective treatment for tRCC and there are no biomarkers of response to treatments in these patients. We present a case of a 23 year-old female with metastatic tRCC to the lungs who was started on treatment with nivolumab and ipilimumab. She had a complete radiographic response to treatment and has been progression-free for over 18 months. Immunofluorescence imaging performed on the baseline primary tumor sample showed significant intratumoral immune infiltration. Importantly, these cells are present in niches characterized by TCF1+ CD8+ T cells. Histopathologic investigation showed the presence of lymphocytes in the fibrovascular septae and foci of lymphovascular invasion. Furthermore, lymphovascular invasion and intratumor niches with TCF1+ CD8+ T cells may predict a favorable response to treatment with nivolumab and ipilimumab. These findings have significant clinical relevance given that immune checkpoint inhibitors are approved for several malignancies and predictive biomarkers for response to treatment are lacking. Importantly, the identification of these TCF1+ CD8+ T cells may guide treatment for patients with tRCC, which is a rare malignancy without a consensus first-line treatment option.

## Introduction

Translocation-associated renal cell carcinoma (tRCC) is an uncommon, aggressive subtype of non-clear cell renal cell carcinoma (nccRCC). It was first identified in 2004 with technological advancements in genetic profiling, which showed that the malignancy disproportionately affects young patients and has a very poor prognosis (1). Chromosomal translocations involve TFE3 (Xp11.2) or, much less commonly, TFEB (6p21), and these two variants are commonly grouped and referred to as “MiT Family Translocation RCC” (1). SFPQ-TFE3 tRCC accounts for 20-75% of pediatric RCC and approximately 1-4% of adult RCC, with a slight female predominance at 1.6:1. While agents targeting vascular endothelial growth factor (VEGF) have shown efficacy in retrospective studies, there is no consensus first-line systemic therapy for metastatic tRCC (2). Recent studies have demonstrated similar activity of immune checkpoint inhibitors (ICI) in metastatic tRCC, indicating potential therapeutic benefits of PD-1 and VEGF inhibition for non-clear-cell renal cell carcinoma (nccRCC) (3). There is limited data investigating dual ICI therapy in tRCC. In this report, we present a case of a young female with tRCC who experienced an exceptional response on nivolumab and ipilimumab combination therapy. We also present immunofluorescence (IF) imaging and histopathologic staining of the patient’s primary tumor specimen, wherein we specifically probe for the presence of TCF1+ CD8 T cells. The importance of these TCF1+ CD8 T cells for the endogenous immune response to RCC has been demonstrated (4), and others have reported a critical role for these TCF1+ cells in the response to ICI in other tumor types (5–9), but the role of these cells in the response to ICI has been suggested (10), but not definitively established in RCC, particularly in rare subsets of RCC such as tRCC.

## Methods

### Patients and Clinical Data

We collected clinical data for one patient with TFE3-SFPQ fusion tRCC from the electronic medical record. Data regarding histologic diagnosis was obtained from the pathology report and PD-L1 status was determined from a Foundation One immunohistochemical (IHC) test. Radiographic response rate was determined using response evaluation criteria in solid tumors version 1.1 (RECISTv1.1) (11).

### Sample Collection, Preparation, and Storage

Samples for immunofluorescence (IF) analysis were formaldehyde fixed and embedded in paraffin (FFPE) blocks by Emory Pathology. Unstained stained sections of FFPE blocks were obtained from Emory Pathology.

### Deparaffinization and Antigen Retrieval

Sections were deparaffinized in successive incubations with xylene and decreasing concentrations (100, 95, 75, 50, 0%) of ethanol. Antigen retrieval was achieved using Abcam 100x TrisEDTA Antigen Retrieval Buffer (pH = 9) heated to 115°C under high pressure. Sections were washed in PBS + 0.1% Tween20 before antibody staining.

### Immunofluorescence Antibody Staining

Immunofluorescence staining and analysis was performed in a research laboratory at Emory University. Sections were blocked for 30 min with a 10% goat serum in 1x PBS + 0.1% Tween20. Sections were then stained with appropriate primary and secondary antibodies. Primary antibodies were used at concentrations of 1:100 (MHC-II) or 1:150 (CD8, TCF1) and incubated for 1 h at room temperature (**Supplemental Table 1** **)**. Secondary antibodies were used at concentrations of 1:250 (A488, A568) or 1:500 (A647) and incubated for 30 min at room temperature.

### Image Capture and Analysis

The selected fluorophore panel allowed for simultaneous visualization of three targets and a nuclear stain (DAPI). A For Leica SP8 confocal microscope with a motorized stage was used for tiled imaging, and a 40x, 1.3NA, 0.24 mm WD oil immersion objective was used, allowing for highly resolved, smoothly tiled images. Fluorophores were excited with the 496, 561, and 594 laser lines or with a multiphoton Coherent Chameleon Vision II laser, tuned to 700nm (DAPI). Emission-optimized wavelength ranges informed specific detector channels, which were used to detect fluorescence. Leica LASX software was used to create a maximum projection image, allowing for acquisition of large, tiled images regardless of a varying focal plane across each tissue section.

## Results

### Case Presentation and Histologic Description

A 23-year-old female patient presenting with right-sided flank pain underwent a right radical nephrectomy and was diagnosed with a pT1bNxM1 melanotic translocation-associated renal cell carcinoma with TFE3-SFPQ fusion. Caris report found no significant mutations for clinical biomarkers and patient physical exam had no clinically significant findings. A CT scan of the chest showed numerous bilateral lung nodules concerning for metastasis. Four months later, the patient was started on dual ICI therapy with ipilimumab 1mg/kg and nivolumab 3 mg/kg given the patient’s young age and the possibility of a durable radiographic response to immunotherapy, which has been seen in RCC patients treated with ICI-based treatment regimens. She received 4 cycles of dual therapy and has now received 22 cycles of nivolumab 480 mg q28d monotherapy. She experienced diffuse myalgias and arthralgias starting after cycle 5 of treatment which improved with 5mg prednisone. She also experienced adrenal insufficiency requiring hydrocortisone and hypothyroidism controlled on levothyroxine. Although the myalgias, arthralgias, adrenal insufficiency, and hypothyroidism were deemed as likely related to her ICI-regimen, no dose adjustments were required. She otherwise endorsed favorable tolerance with her therapy and felt positively about her treatment course. Her first restaging chest CT scan after cycle 4 showed a complete response, which has been maintained for over 18 months. At the time of this report, the patient is alive and well.

### Correlative Data

Outside PD-L1 IHC staining identified a tumor proportion score of 1%. A Caris molecular intelligence tumor profiling report showed a low TMB, stable MSI, and no detected mutations in BAP1, FH or PBRM1. Germline testing was negative.

### Pathologic Description

**Figure 1:**
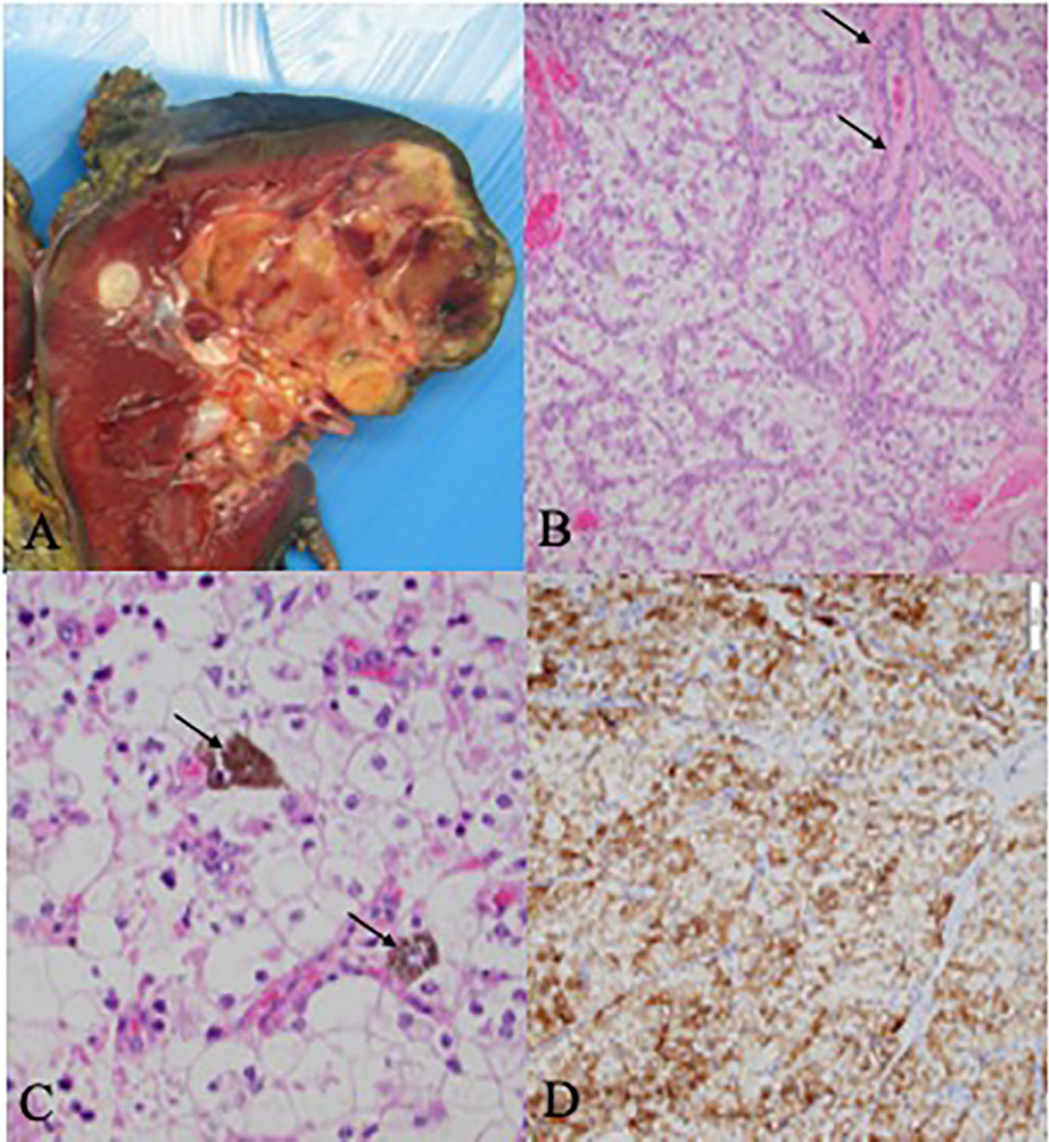
Pathology and Histology Samples **(A)** Gross kidney specimen demonstrating a superior pole multi-nudular, well-defined yellow cream mass with areas of hemorrhagic degeneration and necrosis. **(B)** Histologic examination shows solid nests of polygonal clear cells with prominent neclei with nucleoli. The intervening fibrovascular septae contain small lymphocytic infiltrates (black arrows). **(C)** Unique intra-cytoplasmic melanin pidment which may be seen in melanotic translocation-associated renal cell carcinoma (black arrows). **(D)** The carcinoma was positive for HMB45, a melanocytic marker which can be positive in melantonic translocation-associated renal cell carcinoma.

The right nephrectomy specimen included a well-defined nodular mass confined to the renal parenchyma measuring 5.7 x 4.6 x 4.3 cm (**Figure 1A**). The renal mass showed a relatively homogenous microscopic appearance composed of nests of optically clear polygonal cells separated by fibrovascular septae which contained occasional small reactive lymphocytic inflammation (**Figure 1B**). Some cells contained fine dark brown intracytoplasmic melanin pigment (**Figure 1C**), and the tumor was positive for HMB45, a melanocytic marker (**Figure 1D**). Foci of multinucleated tumoral cells consistent with a WHO/ISUP nuclear grade 4 were present. There were also foci of lymphovascular invasion present.

### Immunofluorescence Imaging

**Figure 2:**
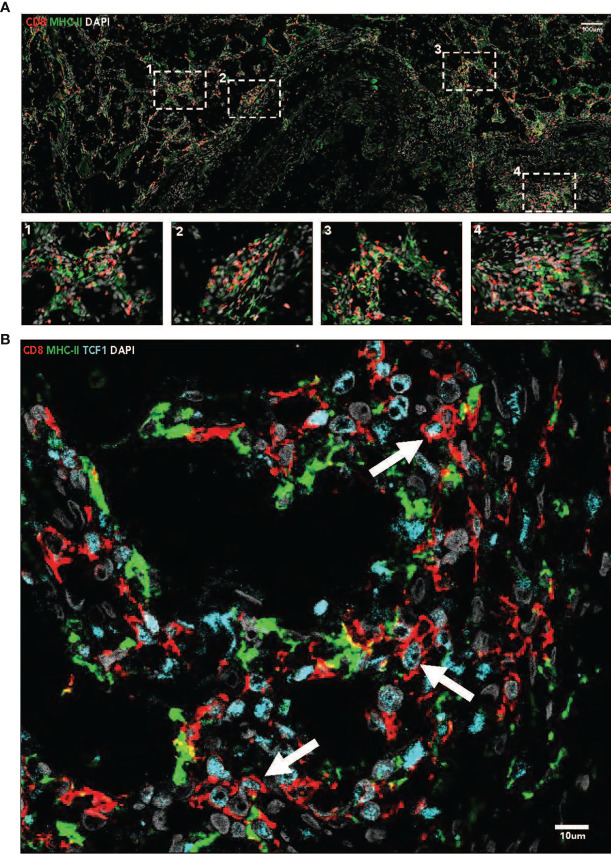
**(A)** Immunofluorescence imaging (40x, tiled) demonstrates the presence of intratumoral immune niches containing CD8+ and MHC-11+ cells. Nuclei are stained with DAPI. **(B)** Immunofluorescence imaging (40x) demonstrates the presence of TCF 1+ CD8+ T cells in MHC-11+ antigen presenting cell dense niches. Nuclei are stained with DAPI.

Immunofluorescence imaging reveals presence of CD8+ and MHC-II+ cells in tumor tissue. CD8+ T-cells are found throughout the tumor issue, as well as in dense aggregations, or immune niches, with MHC-II+ antigen presenting cells (**Figure 2A**). Many CD8+ T-cells found in these niches are TCF1+ (**Figure 2B**).

## Discussion

In this report, we present a case of a young woman with metastatic TFE3-SFPQ fusion translocation RCC who had a durable complete response on nivolumab and ipilimumab combination therapy. Targeted therapy with mTOR or VEGF inhibitors was also under consideration based on clinical trials showing efficacy in tRCC (2, 12, 13). However, ICI-based therapy was selected as first-line therapy for this young woman based on the promise of a durable response (14, 15), especially in the context of recent reports suggesting promising activity for ICIs in nccRCC, and specifically tRCC (14, 16, 17). Ongoing trials continue to explore the efficacy of immune checkpoint inhibitors in tRCC (16).

We showed that intratumoral immune niches were present in the primary tumor specimen, which were characterized by TCF1+ CD8+ T-cells. This was supplemented by the histologic finding of the presence of lymphovascular invasion and lymphocytic infiltration in fibrovascular septae between tumor cells. These correlations have significant clinical implications because: (1) there is no consensus standard-of-care treatment for tRCC, (2) there are no validated predictive biomarkers of response to ICI in tRCC, and (3) these findings may be generalizable to other rare, aggressive malignancies which ICI are not currently FDA approved for if validated in larger, prospective studies.

Non-clear cell RCC is a heterogeneous group of malignancies that have no consensus standard-of-care treatments. A few small, retrospective studies have suggested VEGF inhibitors may have efficacy for treatment of metastatic tRCC, identifying similar PFS and objective responses compared to patients with clear-cell RCC (2). Given the high recurrence rates in tRCC patients treated with targeted therapy or chemotherapy, immunotherapy is an attractive option for these young patients, particularly given that these agents offer the promise of a possible durable response. This is particularly true for tRCC, which disproportionately affects younger patients and has an aggressive course and a very poor prognosis. Recent data has suggested that ICIs have efficacy in treating nccRCC, including tRCC (14, 17). Given that there is limited data on treatment options for tRCC patients, this report has significant clinical value for medical oncologists because it provides a possible predictive marker of response to ICI and a potential mechanism by which patients with rare RCC histology respond to ICI. Furthermore, it is unclear whether specific subtypes of tRCC are more likely to respond to ICI and this report provides hypothesis-generative data regarding the sensitivity of TFE3-SFPQ fusion tRCC to ICI.

There has been an expanding effort to identify biomarkers of response to ICI given their increasing prevalence in the treatment of many malignancies including RCC. Miao et al. identified an association between loss of the PBRM1 gene and clinical outcomes, arguing for absence of this gene as a prognostic marker for improved response to ICI-based regimens (18). Although PD-L1 expression has value as a predictive biomarker in some solid tumors, there is no clear association in RCC patients treated with ICI (19). The limitation of PD-L1 expression as a biomarker is highlighted in our patient’s case, as the IHC score was 1% and, despite this low expression, the patient experienced a complete response to treatment. A potential contributing factor could have been the degree of T-cell infiltration in the tumor’s histology, which is a finding that has been associated with improved patient outcomes in many tumor types and clinical settings (20–25), and specifically in RCC patients receiving immunotherapy (24).

Subsequent study has suggested that TCF1+ CD8 T cells are the subset of the tumor infiltrating lymphocytes that are critical for the response to ICI in those tumors where CD8 T cell infiltration predicts patient outcomes (5–9). TCF1 is a transcription factor which defines a stem-cell like population of CD8+ T cells, and furthermore these cells have demonstrated increased proliferative potential, expression of survival-related genes such as IL7R, and generation of co-stimulatory molecules such as CD28 when compared to CD8+ T cells without TCF1 (4–9). In other tumor types, such as melanoma, the number of these TCF1+ CD8 T cells correlates with therapeutic response. This suggests that the presence of intratumoral TCF1+ CD8 T cells are critically important to the response to ICI in several tumor types (4–9). Thus, in the context of the findings that these TCF1+ stem-cell like CD8+ T cells reside in immune niches with a high density of antigen presenting cells in RCC (4), we looked for the presence of these cells in these dense immune niches in this patient with tRCC, given the patient’s exceptional response.

Interestingly, these cells were identified in the baseline tumor sample of the patient presented in this report and appeared in close association with MHC-II+ antigen presenting cells, similar to what was reported in Jansen et al. (4). Additionally, lymphocytes were identified in the fibrovascular septae with foci of lymphovascular invasion on histopathologic investigation. This hypothesis-generating data may suggest that further study of these TCF1+ stem-like T cells and antigen presenting niches may reveal a prognostic and predictive biomarker for tRCC patients treated with ICI, especially given that recent reports have associated TCF1 enrichment with response to ICI in other RCC subtypes (10). While this case report is only a preliminary view into the role of TCF1+ CD8 T cells in the response to tRCC, and in RCC in general, it provides an interesting and important foundation for future, more comprehensive study into these cells as both a biomarker of and as a key mechanistic player in the response to ICI in RCC.

The strength of this case report is that it offers a unique anecdote of the potential for combination ICI therapy in patients with tRCC and highlights the potential for the presence of TCF1+ CD8+ T cells as a biomarker in this rare malignancy. Further investigation is needed regarding the potential predictive and prognostic of this biomarker to predict responses to immunotherapy. The main limitation of this study is that this is a case report which limits the generalizability of this finding without validation in larger, prospective studies. Additionally, the relatively short follow-up including in this case report limits our ability to assess the durability of the patient’s response. However, as of October 2021, over 18 months after the initiation of therapy, the patient has maintained a complete response on follow-up imaging studies.

## Data Availability Statement

The original contributions presented in the study are included in the article/**Supplementary Material**. Further inquiries can be directed to the corresponding author.

## Ethics Statement

Written informed consent was obtained from the individual(s) for the publication of any potentially identifiable images or data included in this article.

## Author Contributions

DM, CJ, and LH drafted the manuscript. DM collected the clinical data for the patient included in this study and provided administrative support. CJ provided the immunofluorescence images and LH provided the histopathologic images. MB oversaw the study. VM was the urologic surgeon that performed the patient's nephrectomy and provided samples used in the biopsy slides. All authors edited the manuscript and approved the final version of the manuscript.

## Funding

Research reported in this publication was supported in part by the Breen Foundation and the Biostatistics Shared Resource of Winship Cancer Institute of Emory University. The content is solely the responsibility of the authors and does not necessarily represent the official views of the National Institutes of Health.

## Conflict of Interest

MB has acted as a paid consultant for and/or as a member of the advisory boards of Exelixis, Bayer, BMS, Eisai, Pfizer, AstraZeneca, Janssen, Calithera Biosciences, Genomic Health, Nektar, and Sanofi and has received grants to his institution from Xencor, Bayer, Bristol-Myers Squibb, Genentech/Roche, Seattle Genetics, Incyte, Nektar, AstraZeneca, Tricon Pharmaceuticals, Genome & Company, AAA, Peloton Therapeutics, and Pfizer for work performed as outside of the current study.

The remaining authors declare that the research was conducted in the absence of any commercial or financial relationships that could be construed as a potential conflict of interest.

## Publisher’s Note

All claims expressed in this article are solely those of the authors and do not necessarily represent those of their affiliated organizations, or those of the publisher, the editors and the reviewers. Any product that may be evaluated in this article, or claim that may be made by its manufacturer, is not guaranteed or endorsed by the publisher.

## Supplementary Material

Supplementary Table 1 Immunofluorescence Antibodies
