# Supplementary material for: Case Report: Exceptional Response to Nivolumab Plus Ipilimumab in a Young Woman With TFE3-SFPQ Fusion Translocation-Associated Renal Cell Carcinoma
Source: Front Oncol. 2021 Dec 16;11:793808. doi: 10.3389/fonc.2021.793808 (PMC8716393; doi:10.3389/fonc.2021.793808)
Supplement: Supplementary Table 1 — Immunofluorescence Antibodies [file Table_1.docx]

**Supplemental Table 1: Immunofluorescence Antibodies**

| **Target** | **Antibody Type** | **Clone** | **Concentration** | **Secondary** | **Concentration** |
| --- | --- | --- | --- | --- | --- |
| MHC-II (HLA-DR, DP, DQ) | Mouse IgG2a | Tu39 | 1:100 | Goat anti-mouse IgG2a A488 | 1:250 |
| TCF1 | Rabbit | C63D9 | 1:150 | Goat anti-rabbit A568 | 1:250 |
| CD8 | Mouse IgG1 | C8/144B | 1:150 | Goat anti-mouse IgG1 A594 | 1:500 |
