# Supplementary material for: Case Report: Exceptional Response to Nivolumab Plus Ipilimumab in a Young Woman With TFE3-SFPQ Fusion Translocation-Associated Renal Cell Carcinoma
Source: Front Oncol. 2021 Dec 16;11:793808. doi: 10.3389/fonc.2021.793808 (PMC8716393; doi:10.3389/fonc.2021.793808)
Supplement: Supplementary file 2 [file DataSheet_1.pdf]

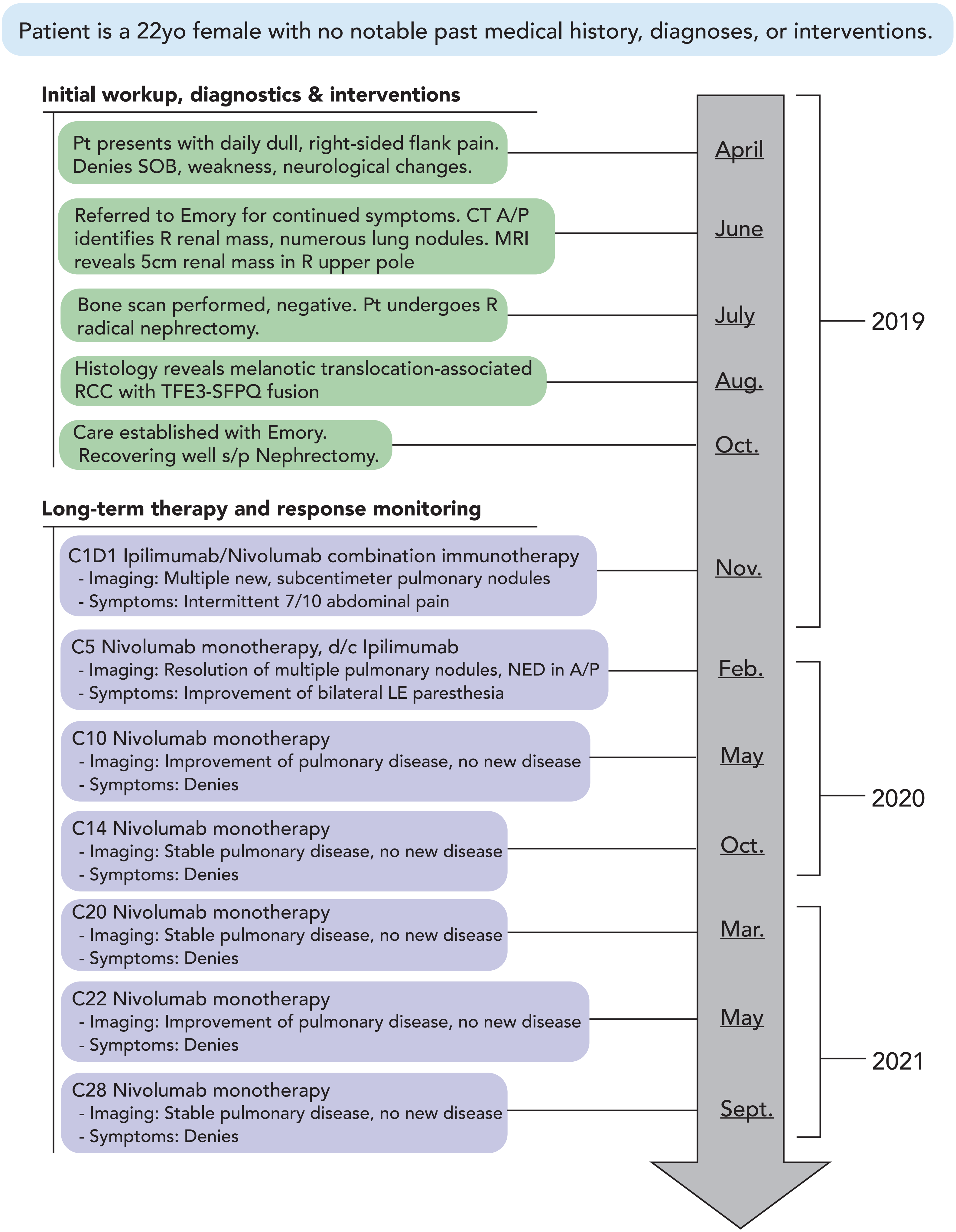
Patient is a 22yo female with no notable past medical history, diagnoses, or interventions.
Initial workup, diagnostics & interventions
Pt presents with daily dull, right-sided flank pain. Denies SOB, weakness, neurological changes.
April
Referred to Emory for continued symptoms. CT A/P identifies R renal mass, numerous lung nodules. MRI reveals 5cm renal mass in R upper pole
June
Bone scan performed, negative. Pt undergoes R radical nephrectomy.
July
Histology reveals melanotic translocation-associated RCC with TFE3-SFPQ fusion
Aug.
Care established with Emory. Recovering well s/p Nephrectomy.
Oct.
2019
Long-term therapy and response monitoring
C1D1 Ipilimumab/Nivolumab combination immunotherapy
- Imaging: Multiple new, subcentimeter pulmonary nodules
- Symptoms: Intermittent 7/10 abdominal pain
Nov.
C5 Nivolumab monotherapy, d/c Ipilimumab
- Imaging: Resolution of multiple pulmonary nodules, NED in A/P
- Symptoms: Improvement of bilateral LE paresthesia
Feb.
C10 Nivolumab monotherapy
- Imaging: Improvement of pulmonary disease, no new disease
- Symptoms: Denies
May
C14 Nivolumab monotherapy
- Imaging: Stable pulmonary disease, no new disease
- Symptoms: Denies
Oct.
2020
C20 Nivolumab monotherapy
- Imaging: Stable pulmonary disease, no new disease
- Symptoms: Denies
Mar.
C22 Nivolumab monotherapy
- Imaging: Improvement of pulmonary disease, no new disease
- Symptoms: Denies
May
C28 Nivolumab monotherapy
- Imaging: Stable pulmonary disease, no new disease
- Symptoms: Denies
Sept.
2021
